# Exploring the impact of macrophage polarization and Modic changes on baseline clinical condition in radiculopathy due to lumbar disc herniation

**DOI:** 10.1016/j.bas.2025.104388

**Published:** 2025-08-06

**Authors:** Wensen Li, Niek Djuric, Christiaan Mink, Carmen L.A. Vleggeert-Lankamp

**Affiliations:** a Neurosurgical Center Holland, Leiden University Medical Center & Haaglanden Medical Center & Haga Teaching Hospital, the Netherlands; b Erasmus MC, Rotterdam, the Netherlands; c Spaarne Gasthuis, Hoofddorp, Haarlem, the Netherlands

**Keywords:** Sciatica, Lumbar, Inflammation, Macrophage, M1 and M2, Modic changes

## Abstract

**Introduction:**

Lumbar intervertebral disc herniation (LDH) often manifests as sciatica, resulting from mechanical compression and/or an inflammation affecting the nerve root.

**Research question:**

This study aims to investigate the impact of inflammation and macrophage polarization on clinical symptoms in surgical LDH patients.

**Material and methods:**

Nucleus pulposus (NP) tissue samples were obtained from LDH patients. Clinical symptoms were assessed using the Numerical Rating Scale (NRS) for back pain and leg pain, and the Oswestry Disability Index (ODI). HE and CD68 staining were employed to detect inflammation in the samples while CD163 and CD192 were utilized as markers for M1 and M2 macrophage phenotypes respectively. MRI was screened for Modic changes (MC).

**Results:**

A total of 187 patients were included. Inflammation was found in 140 patients, with 93 having severe inflammation. MC type I was present in only 8 % of patients (16 patients), type II in 37 %. Inflammation severity correlated with back pain (p = 0.021). M1 macrophage dominance associated with the presence of more intense back pain, especially in patients with MC type II (p = 0.001). Inflammation severity positively correlated with leg pain, but only in patients without Modic changes (p = 0.003).

**Discussion and conclusion:**

The link between back and leg pain and intervertebral disc inflammation appears to be influenced by the presence or absence of MC. These findings reveal distinct roles for M1 and M2 macrophages in clinical symptoms, warranting further investigation. Large-scale cohort studies are needed to validate and expand upon these observations.

## Highlights

• Type I Modic changes and back pain are likely linked with M1 macrophage-dominant inflammation, requiring further validation.
• Leg pain was more strongly associated with significant inflammation in patients without Modic changes.
• Promoting M1-to-M2 macrophage shifts and anti-inflammatory therapy may treat pain from Modic changes and disc herniation.
• Type I Modic changes may serve as a biomarker of M1 macrophage-driven inflammation and help guide targeted treatment.

## 1 Introduction

Lumbar disc herniation (LDH) is a common condition characterized by the displacement of nucleus pulposus (NP) material beyond the disc space, often resulting in compression of adjacent neural structures and the onset of low back pain and radiating symptoms (Rajasekaran et al., 2013). An important aspect of the pathophysiology of LDH revolves around the inflammatory response induced by herniated disc material within the epidural space, in particular the infiltration of macrophages (Djuric et al., 2020a). It is hypothesized that the herniation of NP material triggers a foreign body reaction, which induces macrophage infiltration into the affected area (Djuric et al., 2019). While these macrophages are thought to participate in the resorption of herniated disc material, they also contribute to the induction of an inflammatory cascade, potentially exacerbating pain symptoms. The role of macrophages in LDH-induced inflammation is still controversial, with different opinions based on the different phenotypic characteristics of macrophages in the disc environment.

The current literature distinguishes between two main phenotypes of macrophages: M1 and M2. M1 macrophages, stimulated by factors such as lipopolysaccharide (LPS), interferon-gamma (IFN-γ) and tumor necrosis factor (TNF), exhibit a pro-inflammatory profile characterized by the secretion of cytokines such as IL-1, IL-6 and TNF-α. These pro-inflammatory mediators have been implicated in nociceptive sensitization and the exacerbation of pain symptoms associated with LDH (Martinez et al., 2014; Arango Duque et al., 2014; Dube et al., 2017; Pedersen et al., 2015). Conversely, M2 macrophages, induced by factors such as IL-4, IL-10 and glucocorticoids, have an anti-inflammatory profile and secrete cytokines such as IL-10 and transforming growth factor-beta (TGF-β), which promote tissue repair and remodeling and may alleviate pain symptoms by resorbing herniated disc material (Martinez et al., 2014; Zhang et al., 2020). Despite numerous studies, the exact role of M1 and M2 macrophages in LDH-related inflammation and pain remains unclear. A review by Djuric et al. (2020b) identified that M1-related cytokines, such as TNF-α, TNFR1, IL-6, IL-8, and IFN-γ, were associated with higher pain scores, while M2-related cytokines, including IL-4 and IL-10, were linked to lower pain scores. This finding aligns with the hypotheses mentioned above.

The presence of Modic changes (MC) on MRI, also referred to as vertebral endplate signal changes (VESCs), has been widely investigated in relation to low back and leg pain. While several studies have found no consistent association between MC and pain symptoms in patients with lumbar disc herniation (el Barzouhi et al., 2014), and that patients with MC may show poorer response to conservative treatment without clear improvement in pain or herniation size (Shan et al., 2014). In contrast, other research has suggested that MC may be involved in pain progression. For instance, Modic changes were reported to be an important covariate in the association between reduction in leg pain and the presence of macrophages in disc material in patients operated for symptomatic lumbar disc herniation (Djuric et al., 2019). Modic type I changes have been shown to represent an active inflammatory state within the vertebral endplate and adjacent bone marrow (Dudli et al., 2017; Albert et al., 2013a). Given the central role of macrophages in the regulation of inflammation, the hypothesis that different macrophage phenotypes (particularly M1 and M2) may have a potential correlation on the pathophysiology of Modic changes was raised. We therefore performed a pilot study to evaluate the M1 and M2 characterization in disc material in the presence/absence of MC. We could confirm that higher levels of CD68^+^ in symptomatic lumbar disc herniations associated with MC, indicating an association with the presence of macrophages. Moreover, we demonstrated a relatively high expression of M2 markers if MC were absent, in accordance with the theory that M2 macrophages have a regulatory role in tissue repair and anti-inflammatory responses (Djuric et al., 2019). Furthermore, in evaluating the association between MC presence and type and macrophage presence and type, we observed that if MC type I changes were present, the occurrence was highly associated with both severe (p = 0.016) and M1 macrophage-dominant inflammation (p = 0.048).

Based on previous studies, it is reasonable to hypothesize that more severe disc inflammation and M1 type macrophages dominated inflammatory milieu will be associated with more pronounced back and radicular pain, and that the different types of Modic changes are clinical biomarkers that signify a certain type of inflammation in the disc. The purpose of the current study was to investigate the association between disc inflammation, macrophage polarization, and the clinical condition at baseline in patients undergoing surgery for LDH. As baseline data avoids confounding by post-surgical changes, allowing isolation of inflammatory effects. Specifically, we aimed to (1) investigate whether the severity of disc inflammation, the predominance of M1 or M2 macrophages in herniated disc tissue correlate with the intensity and distribution of pain; (2) assess whether the presence and type of MC is of influence on these associations. With this study, we expect to elucidate the interplay between local inflammation and Modic changes in symptomatic LDH, in order to provide a theoretical basis for more personalized treatment of patients with radiculopathy due to lumbar disc herniation in the future.

## 2 Methods

### 2.1 Study design

A multicenter, prospective observational cohort study was conducted among patients with lumbosacral radiculopathy due to a herniated disc, confirmed by MRI evaluation. The protocol was approved in all four participating centers by the Medical Ethics Committee of the Leiden University Medical Center, and subsequently by the Board of Directors of the Alrijne Hospital Leiderdorp/Leiden, Spaarne Gasthuis Haarlem/Hoofddorp and the HAGA hospital the Hague (P18.211). Written informed consent was obtained from all patients. Details of the protocol have been published previously (Djuric et al., 2021). This trial was registered with the Netherlands Trial Register (no. NL8464, www.trialregister.nl).

### 2.2 Participants

Patients were eligible if they fulfilled the inclusion criteria: age between 18 and 75 years old, presence of a radiologically (MRI) proven herniated disc consistent with clinically relevant lumbar radiculopathy, patient to be eligible and scheduled for lumbar discectomy, and presence of disabling clinical symptoms for more than 8 weeks (Djuric et al., 2021). Exclusion criteria were: previous lumbar spinal surgery, loss of strength in the leg (MRC <4), history of spinal inflammatory disease, instability requiring instrumented spondylodesis surgery, active infection at the time of surgery, and use of antibiotics in the six months preceding surgery. Other exclusion criteria were short-term planned migration, no or limited understanding of the Dutch language, and pregnancy.

### 2.3 Intervention

Unilateral transflaval microdiscectomy was performed in all patients. The level of the incision was determined using fluoroscopy. After a small midline incision, the muscle was detached from the spinous processes. A small horizontal hemilaminectomy at the side of the hernia was performed and the ligamentum flavum was reduced. Removal of the herniated disc material was performed and continued until the nerve root was completely decompressed. The resected disc material was collected and stored in 4 % formaldehyde and within hours transported to the LUMC pathology laboratory.

### 2.4 Immunohistochemistry

The harvested disc tissues were fixed in 4 % formaldehyde solution for 3–7 days. Tissue was subsequently embedded in paraffin blocks and 5-μm thick slices were taken from the middle of the block for haematoxylin and eosin staining, which was performed according to the Leica ST 5020-mulitstainer standard protocol. Samples were evaluated under a microscope for the presence of inflammatory cells. If tissue from one sample exceeded the capacity of 1 paraffin block, multiple blocks were formed, and a slide of each block was evaluated.

In case of positive HE stained cells, additional slices were prepared for immunohistochemistry staining. For the staining procedure, three serially cut 5-μm paraffin slices were rinsed in ethanol and methanol solutions and prepared for the expression of CD68 (1:250; to visualize macrophages; DAKO Denmark), CD192 (1:1000; to visualize macrophage type 1: M1; Thermo Fisher Scientific, Netherlands), and CD163 (1:600; to visualize macrophage type 2, M2; Abcam, Netherlands) respectively. Immunohistochemistry was performed using a three-step indirect method. Antibodies CD68 were cooked in Citrate pH 6.0 buffer, CD 192 and CD 163 were cooked in EDTA pH 8.5 buffer as a pre-treatment. Primary antibodies were incubated overnight in a humid chamber at room temperature and secondary antibodies were incubated for 1 h. Subsequently, an avidin-biotin complex technique was performed with the Vectastain ABC-Elite Kit (Vector Lab. USA) and the appropriate biotinylated antibodies. Visualization of the peroxidase reaction was done with DAB solution (Sigma). Samples were counterstained with Harris haematoxylin. All samples were accompanied by a positive control, which was atherosclerosis tissue for all macrophage markers (Djuric et al., 2021). For evaluation all samples were photographed using Philips ultra-fast scanner.

### 2.5 Macrophage counting

Cell counts were performed using ImageJ and evaluation was executed by two independent researchers (ND & WL). Inter-observer correlation coefficients were calculated for each staining separately. For each antibody 50 pictures were evaluated by hand: cells were analyzed based on morphological features and only macrophages were photographed and evaluated. Subsequently an automated cell count algorithm in ImageJ was matched on the average count of the two observers with a correlation coefficient of >0.8, which was regarded as a strong correlation. This algorithm and parameter settings were used for evaluation on all images. Positive macrophage/lymphocyte counts were divided by the surface of the evaluated herniated disc material in cm^2^.

**Fig. 1:**
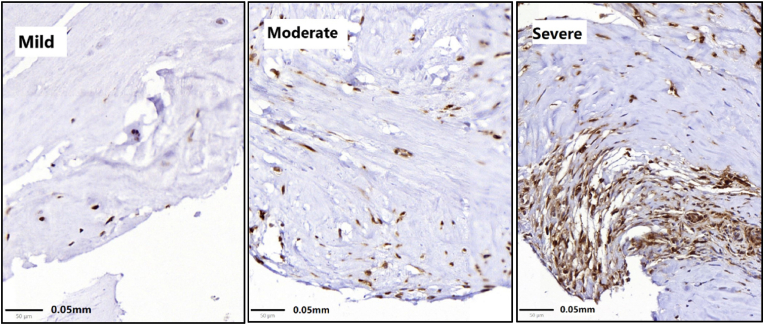
Examples of mild (0–10 macrophages per cm^2^), moderate (10–100 macrophages per cm^2^), and severe (>100 macrophages per cm^2^) inflammation.

Firstly, samples were evaluated for the presence of HE positive cells, to determine whether inflammatory cells were present. Thereafter the samples were evaluated based on the presence of CD68 staining and categorized based on our previous study, in which a logarithmic scale was chosen (Djuric et al., 2019): no inflammation (0 macrophages; including the samples that did stain negative with the HE staining), mild (1–10 macrophages per cm^2^), moderate (10–100 macrophages per cm^2^) and severe (>100 macrophages per cm^2^) inflammation (Fig. 1). Dichotomous inflammation grouping: minimal inflammation (0–10 macrophages per cm^2^) and considerable inflammation (>10 macrophages per cm^2^).

Subsequently, M1 and M2 dominance was evaluated in the samples containing CD68 positive cells: it was only determined if at least 10 positive CD68 cells/cm^2^ were present. For relative M1/M2 dominance, M1 and M2 markers were expressed as percentages (ranging from 0 % to 100 %) of the number of CD68 positive cells. M1 dominance was defined when the percentage of M1 cells counts was more than 60 %. If the percentage of M1 was between 40 % and 60 %, it is defined as M1 equal to M2. M2 dominance was defined if the percentage of M1 cell count was less than 40 %.

### 2.6 Modic change evaluation

MRI scans were performed at baseline by a 1.5 or 3.0 T scanner, and both sagittal T1-and T2-weighted images of the lumbar spine were evaluated. MRIs were evaluated by 2 investigators (WL & ND), blinded for the clinical condition, and the interobserver agreement was evaluated using Cohen's kappa coefficient. All cases were the two investigators disagreed; final verdict was supplied by a third reviewer: a senior neurosurgeon with over 20 years of experience in spine care (CVL) to reach final consensus. All three were blinded to histological data and clinical information. The readers were not involved in the selection or treatment of the patients included. Modic changes were assessed and classified as present or absent, and scored as Type I, Type II, or Type III, according to the criteria of Modic et al., 1988a, 1988b.

### 2.7 Clinical evaluation

Patients were recruited during their initial visit to the neurosurgeon. After informed consent was obtained patients completed online questionnaires covering demographics (age, gender, BMI etc.), back and leg pain scores, and disability scores. Additionally, the percentages of patients with significant pain (NRS >4) and significant disability (ODI >24) will be estimated using previously published cut off values (Mjåset et al., 2020).

NRS back pain: patients indicate the amount of pain they have experienced during the week previous to the visit. The pain intensity is determined on a scale of 0–10.0 represents ‘no back pain’ and 10 represents ‘worst back pain imaginable’.

NRS leg pain: patients indicate the amount of pain they have experienced during the week previous to the visit. The pain intensity is determined on a scale of 0–10.0 represents ‘no leg pain’ and 10 represents ‘worst leg pain imaginable’.

Oswestry Disability index (ODI): disability is scored with 10 topics related to the impact of lumbar radiculopathy on the patient's life, with 5 gradings for each topic. The total will give a score between 0 (no disability) and 50 (maximum disability possible), which will be calculated to a 1–100 % score (Djuric et al., 2021; Haefeli et al., 2006).

### 2.8 Data analysis

For continuous variables such as age, BMI, symptom duration, and pain scores: t-tests were used for comparisons between two groups, ANOVA was used for comparisons between three groups for normally distributed data, and Kruskal-Wallis tests were used for non-normally distributed data. Correlations between inflammation severity, macrophage dominance groups and Modic changes (MC) with the binary categorization of baseline values were assessed using the chi-square test (X^2^-test). In analyzing the correlation between inflammation and MC types, as well as the correlation between the inflammation severity or macrophage type dominance groups under different MC types and the baseline binary values, Fisher's exact test was employed when the frequency of certain groups was less than 5.

## 3 Results

### 3.1 Characteristics of the study population

A total of 237 consecutive patients scheduled for lumbar herniated disc surgery were assessed eligible for inclusion between June 2019 and October 2023. After screening according to the exclusion criteria, a total of 187 patients, with a mean age of 47 years enrolled in the study (Table 1).

**Table 1:** Overview of clinical condition, immunohistological results and MC ∗ P-value <0.05

	Total	No MC	MC	MC I	MC II	P (No MC vs MC)	P (No MC vs MC I vs MC II)
**Age (y)**	47.1 ± 12.3 (N = 187)	46.1 ± 13.0 (N = 102)	48.2 ± 11.3 (N = 85)	50.7 ± 10.6 (N = 16)	47.6 ± 11.5 (N = 69)	0.482	0.335
**Male gender**	44 %	43 %	45 %	50 %	43 %	0.883	0.903
**BMI**	26.5 ± 4.6 (N = 174)	26.8 ± 5.0 (N = 93)	26.0 ± 4.0 (N = 81)	25.4 ± 3.4 (N = 15)	26.2 ± 4.1 (N = 66)	0.287	0.709
**Baseline values**							
**Duration of symptoms (days)**	56.6 ± 121.3 (N = 168)	50.6 ± 95.2 (N = 90)	63.6 ± 146.1 (N = 78)	66.7 ± 75.4 (N = 14)	62.9 ± 157.9 (N = 64)	0.195	**0.034**∗
**NRS back pain**	4.5 ± 2.7 (N = 154)	4.6 ± 2.7 (N = 83)	4.3 ± 2.6 (N = 71)	5.3 ± 2.5 (N = 13)	4.0 ± 2.6 (N = 58)	0.443	0.210
**NRS leg pain**	6.1 ± 2.1 (N = 158)	5.9 ± 2.4 (N = 86)	6.2 ± 1.6 (N = 72)	5.9 ± 2.1 (N = 13)	6.3 ± 1.5 (N = 59)	0.331	0.828
**ODI**	48.4 ± 17.2 (N = 154)	46.1 ± 17.5 (N = 83)	51.1 ± 16.4 (N = 71)	44.2 ± 16.6 (N = 13)	52.7 ± 16.2 (N = 58)	0.072	0.053
**Baseline values (dichotomized)**							
**Backpain+**	62 % (N = 95)	60 % (N = 50)	63 % (N = 45)	85 % (N = 11)	59 % (N = 34)	0.690	0.202
**Legpain+**	90 %(N = 142)	87 % (N = 75)	93 % (N = 67)	85 % (N = 11)	95 % (N = 56)	0.225	0.169
**ODI +**	90 % (N = 139)	88 % (N = 73)	93 % (N = 66)	85 % (N = 11)	95 % (N = 55)	0.296	0.252
**Inflammation severity (CD68)**							
**No staining (0)**	55	29	26	1	25		
**Mild (1**–**10)**	8	4	4	0	4		
**Moderate (10**–**100)**	31	20	11	3	8		
**Severe (>100)**	93	49	44	12	32		
**Macrophages Type**							
**M1 dominant (CD192)**	89	49	40	12	28		
**M1 equals M2 (CD192+CD163)**	28	16	12	2	10		
**M2 dominant (CD163)**	17	10	7	1	6		

### 3.2 Clinical evaluation at baseline

At baseline, mean symptom duration was 57 days ±121. Patients in the MC I group had the longest duration of symptoms (66.7 ± 75.4 days, P = 0.034). Due to incomplete survey responses, the back pain was reported by 154 patients with an average NRS of 4.5 ± 2.7, and 62 % demonstrated an NRS >4. The patients that reported on the leg pain (158 patients) scored an NRS leg pain of 6.1 ± 2.1, with 90 % demonstrating an NRS >4. The ODI at baseline (154 patients) was reported to be 48.4 ± 17.2, with 90 % scoring an ODI >24 (Table 1).

### 3.3 Modic changes

MC at the level of the lumbar disc herniation were present in 85 of 187 (45 %) patients. The interobserver agreement was 82 %, with a kappa value of 0.72. MC type II was identified in 69 of 187 patients (37 %) and MC type I was present in 16 of 187 patients (8 %). No type III Modic changes were observed. There were no significant differences in age, gender and BMI between the overall patient group, the non-MC group and the MC group (Table 1).

### 3.4 Immunohistological data analysis

Evaluation of the HE staining results revealed that inflammatory cells were present in 140 (75 %) of 187 patients (kappa value of 0.94). CD68 staining to identify macrophages showed the following distribution: 55 did not stain positive for macrophages (negative in HE staining and negative in CD 68 staining) (29 %), 8 (4 %) patients were classified as having mild inflammation, 31 (17 %) as having moderate inflammation, and 93 (50 %) as having severe inflammation.

The subsequent evaluation of the CD163 and CD192 characterization of macrophages revealed that two of the samples that stained negative for CD68 antibodies did stain positive for CD163 and CD192, coming to a total of 134 patients out of 187 displaying macrophages. The M1 phenotype predominated in the majority 89 of 134 samples (48 %), type M2 predominated in 17 patients (9 %), and M1 equaled M2 in 28 patients (15 %) (Table 1).

### 3.5 Association between baseline clinical condition and immunohistological results

If patients had relevant back pain it was more likely they had considerable inflammation (p = 0.021, Table 2). Furthermore, when assessing the different types of inflammation, this study indicated that back pain was primarily associated with M1 inflammation, as 51 of the 95 patients with NRS back pain >4 demonstrated M1 macrophages (70 % of the total number of patients demonstrating M1 macrophages), and 18 of the patients with significant back pain demonstrated a mixed type of inflammation (78 % of the patients with M1 = M2 macrophages), whereas 56 % of the patients with M2 dominant inflammation and 40 % of the patients without inflammation reported less back pain (p = 0.005).

**Table 2:** Associations between dichotomized clinical condition and immunohistological results ‘Back pain+’ is defined as NRS≥4; ‘Leg pain+’ is defined as NRS≥4; ‘ODI +’ is defined as ODI≥24; 158 patients filled in the questionnaires on leg pain and 154 patients filled in the questionnaires on back pain and ODI: therefore percentages were also indicated of patients with severe back/leg pain of ODI for each inflammation subgroup. In the no MF group 1 case was missing for ODI, in the M1 dominant group 2 cases were missing for back pain and ODI, for M1 = M2 group, 1 case was missing in for back pain and ODI; ∗ P-value <0.05

	Back pain+ (n = 95) N (%)	Leg pain+ (n = 142) N (%)	ODI+ (n = 139) N (%)
**Inflammation severity**			
**Minimal (N = 63)**	24/50 (48 %)	42/51 (82 %)	44/50 (88 %)
**Considerable (N = 124)**	71/104 (68 %)	100/107 (93 %)	95/104 (91 %)
**p-value**	**0.021**∗	**0.046**∗	0.566

**Macrophage type (N = total)**			
**M1 dominant (N = 89)**	51/73 (70 %)	68/75 (91 %)	66/75 (88 %)
**M1 = M2 (N = 28)**	18/23 (78 %)	23/24 (96 %)	22/24 (92 %)
**M2 dominant (N = 17)**	9/16(56 %)	16/16 (100 %)	15/16 (94 %)
**No MF (N = 55)**	17/43 (40 %)	35/44 (81 %)	36/43 (84 %)
**p-value**	**0.005**∗	0.169	0.252

Likewise, if the patients had relevant leg pain it was more likely they had considerable inflammation (p = 0.046). But leg pain was not clearly associated with either M1 or M2 inflammation. Additionally, if the patients had significant disability (ODI >24) this was not reflected by the severity of inflammation (p = 0.566), nor was there a correlation with the type of macrophages (p = 0.252).

### 3.6 Association between baseline clinical condition and MC

The mean back pain in patients at baseline was 4.5 ± 2.7. Back pain was comparable between patients with (4.3 ± 2.6) and without MC (4.6 ± 2.7; p = 0.443). However, in patients with MC type I back pain was somewhat more severe (5.3 ± 2.5), though not statistically significant (p = 0.210, Table 1). Dichotomising the data demonstrated that 62 % of patients had relevant back pain (NRS >4). This percentage was comparable between patients with (63 %) and without MC (60 %; p = 0.690). The number of patients with MC type I in the studied group of patients was limited (n = 16) and unfortunately, only 13 of them filled in the clinical baseline parameters. In patients with MC type I somewhat more patients had relevant back pain (85 %) than the patients without MC (60 %) or with MC type II (59 %), but again not statistically significant (p = 0.202, Table 1).

The mean leg pain in patients at baseline was 6.1 ± 2.1. Leg pain was comparable between patients with (6.2 ± 1.6) and without MC (5.9 ± 2.4; p = 0.331). In patients with MC type II leg pain is somewhat more severe (6.3 ± 1.5, p = 0.828), though not statistically significant. Dichotomising the data demonstrated that 90 % of patients had relevant leg pain (NRS >4). This percentage was comparable between patients with (93 %) and without MC (87 %; p = 0.225). In patients with MC type II (95 %), leg pain was somewhat more than in patients without MC (87 %) or with MC type I (85 %; p = 0.169) (Table 1).

The mean ODI in patients at baseline was 48.4 ± 17.2. ODI was comparable between patients with (51.1 ± 16.4) and without MC (46.1 ± 17.5; p = 0.072). However, in patients with MC type II ODI was somewhat more severe (52.7 ± 16.2, p = 0.053).

Dichotomising the data demonstrated that 90 % of patients had relevant ODI (ODI >24). This percentage was comparable between patients with (93 %) and without MC (88 %; p = 0.296). In patients with MC type II (95 %), this was somewhat more than in patients without MC (88 %) or with MC type I (85 %; p = 0.252) (Table 1).

### 3.7 Association between immunohistological results and MC

Furthermore, the association between severity of inflammation and MC was evaluated. In agreement with our previous observations (Djuric et al., 2019) an association between severe inflammation and the presence of MC type I was demonstrated: 94 % of patients with MC type I displayed considerable inflammation, in contrast to the patients without MC (68 %) and type II MC (58 %) (p = 0.016; Table 3). Further exploration considering also the macrophage polarization revealed that if MC type I was present, inflammation was most likely of the M1 macrophage type (p = 0.048) (Table 3).

**Table 3:** Associations between immunohistological results and MC Fisher's exact test was used for comparisons between groups; ∗ P-value <0.05

	Considerable inflammation (n = 124) (>10/cm^2^)	M1 dominant (n = 89)	M2 dominant (n = 17)
**Modic changes (N = total)**			
**No MC (N = 102)**	69 (68 %)	49 (48 %)	10 (10 %)
**MC I (N = 16)**	15 (94 %)	12 (75 %)	1 (6 %)
**MC II (N = 69)**	40 (58 %)	28 (41 %)	6 (9 %)
**p-value**	**0.016**∗	**0.048**∗	1.000

### 3.8 Association between baseline clinical condition and immunohistological results in MC subgroups

Finally, the association between baseline clinical parameters and severity of inflammation was studied in MC subgroups. In the subgroup analysis, in patients with MC type I and in patients with MC type II, back pain was dominantly present in patients with considerable inflammation. However, statistically this could only be demonstrated in the MC type II patients (p = 0.006), because too few MC type I patients had minimal inflammation with significant back pain, a reliable Fisher's exact test could not be performed (Table 4).

**Table 4:** Correlation between baseline clinical condition and dichotomized inflammation severity in MC subgroups ‘Back pain+’ is defined as NRS≥4; ‘Leg pain+’ is defined as NRS≥4; ‘ODI +’ is defined as ODI≥24; N = available/total; Fisher's exact test was used for comparisons between groups; ∗ P-value <0.05

	Back pain+ N (%)	Leg pain+ N (%)	ODI+ N (%)
**No MC**			
**Minimal (N = 27/34)**	15 (58 %)	19 (70 %)	21 (81 %)
**Considerable (N = 59/69)**	35 (61 %)	56 (95 %)	52 (90 %)
**P value**	0.811	**0.003∗**	0.274

**With MC I**			
**Minimal (N = 1/1)**	1	1	1
**Considerable (N = 12/15)**	10 (83 %)	10 (83 %)	10 (83 %)
**P value**	N/A	N/A	N/A

**With MC II**			
**Minimal (N = 23/29)**	8 (35 %)	22 (96 %)	22 (96 %)
**Considerable (N = 36/40)**	26 (74 %)	34 (94 %)	33 (94 %)
**P value**	**0.006∗**	1.000	1.000

Leg pain was only associated with considerable inflammation in the group without MC, where only 70 % of the patients with minimal inflammation reported severe leg pain, compared to 95 % in patient with considerable inflammation (p = 0.003) (Table 4).

Unravelling the association between clinical and inflammation in MC subgroups even further, by classifying inflammation into macrophage polarization, revealed that back pain was associated with M1 macrophage dominance in both the MC type I and the MC type II subgroup (Table 5). Again, this could only be demonstrated in a statistically sound way in the MC type II group (p = 0.001). Although there was a significant correlation between leg pain and macrophage dominance in the subgroup without MC, it could not be determined whether it correlated with M1 or M2 dominance (Table 5). There was no significant association between ODI and the severity of inflammation in any MC subgroup.

**Table 5:** Correlation between baseline clinical condition and macrophage type in MC subgroups ‘Back pain+’ is defined as NRS≥4; ‘Leg pain+’ is defined as NRS≥4; ‘ODI +’ is defined as ODI≥24; N = available/total; Fisher's exact test was used for comparisons between groups; ∗ P-value <0.05

	Back pain+ N (%)	Leg pain+ N (%)	ODI+ N (%)
**No MC**			
**M1 dominant (N = 39/49)**	24 (62 %)	37 (93 %)	35 (90 %)
**M1 = M2 (N = 12/16)**	9 (75 %)	12 (100 %)	12 (100 %)
**M2 dominant (N = 10/10)**	6 (60 %)	10 (100 %)	9 (90 %)
**No MF (N = 22/29)**	11 (50 %)	15 (65 %)	17 (77 %)
**p-value**	0.590	**0.006∗**	0.275
**With MC I**			
**M1 dominant (N = 9/12)**	7 (78 %)	8 (89 %)	7 (78 %)
**M1 = M2 (N = 2/2)**	2 (100 %)	1 (50 %)	2 (100 %)
**M2 dominant (N = 1/1)**	1 (100 %)	1 (100 %)	1 (100 %)
**No MF (N = 1/1)**	1 (100 %)	1 (100 %)	1 (100 %)
**p-value**	1.000	0.538	1.000
**With MC II**			
**M1 dominant (N = 25/28)**	20 (80 %)	23 (88 %)	24 (96 %)
**M1 = M2 (N = 9/10)**	7 (78 %)	9 (100 %)	8 (89 %)
**M2 dominant (N = 5/6)**	2 (40 %)	5 (100 %)	5 (100 %)
**No MF (N = 19/25)**	5 (26 %)	19 (100 %)	18 (95 %)
**p-value**	**0.001∗**	0.447	0.815

## 4 Discussion

This study employed histological staining techniques to delineate inflammatory changes in herniating lumbar disc tissue in patients suffering from lumbar radiculopathy who underwent surgical decompression of the nerve root. Previous observations demonstrated a positive association between inflammation, particularly caused by M1 macrophages, and the presence of type I Modic changes on MRI. In the current study we evaluated whether this coincided with the severity of clinical symptoms.

Sixty-two percent of the patients in this group had a back pain score of more than 4 (NRS scale) and it was demonstrated that this coincided with considerable inflammation of the nucleus pulposus tissue. Additionally, it was demonstrated that this inflammation was most likely caused by M1 macrophages, particularly in the patients that displayed Modic Changes on MRI at the target level. This fits to the presumption that Modic changes are caused by vascular insufficiency of the vertebral endplate, leading to back pain if coinciding with M1 macrophages. The small sample size precludes definitive conclusions regarding the association with type I or type II MC.

The absence of strong correlations may be partly explained by the limited variability in symptom severity, as the vast majority of the studied patients presented with clinically relevant leg pain (NRS >4). It was demonstrated though that patients with relevant leg pain had more inflammation than those in which leg pain was less severe, especially in those without Modic changes. Based on the current data we cannot draw conclusions on a dominance of M1 or M2 macrophages in the patients with relevant leg pain.

The current findings indicated that Modic changes were possibly associated with clinical pain, but only in combination with inflammatory properties. It seemed that radicular pain was caused by an inflammatory response and it was hypothesized that an inflammatory response is also responsible for MC observed on MRI. Modic changes may be a morphologic manifestation of disc degeneration and inflammation, whereby the infiltrating macrophages, particularly M1 macrophages, and the cytokines they release were also influencing the pain response of the nerve root. It was likely that M1 and M2 macrophages play each a different role in inflammation of the nerve root and in inflammation of the vascular endplate. This could have explained the discrepancy between MRI findings and clinical symptoms observed in many studies (Lambrechts et al., 2023; Czaplewski et al., 2023; Brinjikji et al., 2015; Tomé-Bermejo et al., 2024).

Our findings aligned with those of previous studies (Djuric et al., 2020b; Karppinen et al., 2021; Dudli et al., 2018) that have demonstrated a positive correlation between inflammation, particularly by M1 macrophages, and Modic type I changes in symptomatic LDH patients. Larger cohorts of patients were necessary to explore macrophage typing in patients with and without back pain within the cohort of patients with leg pain to better understand the association between macrophage types and the presence and classification of Modic changes. It is practically infeasible to retrieve disc tissue from patients without leg pain, as they are rarely subjected to surgery.

These findings may have clinical implications: The observed association between M1 macrophage dominance and Modic Type I Changes suggests a possible link between vertebral inflammation and macrophage-driven immune responses. In the treatment of disc herniation, the primary objective should be the control of inflammation, rather than exclusively focus on structural nerve compression. While this study raises the hypothesis that targeting macrophage polarization, such as promoting a shift from M1 to M2, could represent a therapeutic strategy. This indicates a potential shift in clinical practice towards an inflammation-centered model for the management of sciatica and its accompanied low back pain symptoms. In addition, although Modic Type I changes were associated with M1 dominance in our cohort, their role as a biomarker for inflammatory activity remains speculative and requires validation in prospective studies. The results of this study support the need for further examination of inflammatory pathways in LDH and MC, which may ultimately inform more personalized treatment strategies, including anti-inflammatory therapies, as earlier suggested by the study of H. Alberts (Albert et al., 2013b).

### 4.1 Limitation

This study has limitations that must be considered. The presence of inflammatory cells in staining may not be fully representative of the overall condition of the sample, as the pathological wax block sections may not fully encompass all intervertebral disc tissues in one section. Additionally, the non-normal data distribution and unequal variance may have reduced the statistical power of certain analyses and limited the generalizability of the findings. However, by limiting age as an inclusion criterion, it can be assumed that this effect is limited. Also, due to the categorical nature of our statistical analysis, no correction for possible confounders such as BMI, age, symptom duration, or psychosocial factors known to influence pain were made, which reduces the robustness of our findings. Likewise, kappa value for Modic changes was only substantial and not excellent. Furthermore, not all patients filled in the forms on clinical condition. In particular, analyses involving Modic Type I changes were based on relatively small numbers, which may have reduced the statistical power and reliability of these findings. Notably, no type III Modic changes were observed in our dataset, potentially reflecting their relatively low prevalence in clinical populations. However, this absence could indicate selection bias or limitations in imaging sensitivity. Future studies with larger sample sizes are needed to validate these observations. Moreover, some of the patients were subjected to pain reducing injections with corticosteroids and xylocaine in the months before surgery; metabolic comorbidities, BMI, smoking status or other sources of systemic inflammation were not fully accounted for and may have influenced local immune responses. In addition, although CD192 and CD163 are standard markers for M1 and M2 macrophages, they do not fully reflect functional heterogeneity. In future studies, a more detailed profiling such as cytokine expression would improve characterization. Despite these limitations, the study has several significant strengths. Firstly, it includes 187 patients from multiple centers, which increases the credibility of the data due to its large scale. Secondly, strict standardization measures were implemented for the processing and staining of pathological slides to ensure the accuracy and reliability of the results. Therefore, although this study has limitations, its findings still hold value and provide important clues for further exploration of the pathophysiological mechanisms of lumbar disc disease.

## 5 Conclusion

In conclusion, severity of Inflammation in the herniated disc was associated with higher pain scores, with regard to the type of inflammation; M1 dominant inflammation was most associated with back pain scores and was mostly present in patients with Modic type I Changes. These findings should be interpreted with caution due to limitations such as the small sample size in the Modic type I changes subgroup and the potential influence of unmeasured confounders. To further elucidate the relationship between macrophage types and clinical symptoms in patients with and without Modic changes (type I and II), future prospective studies should combine longitudinal pain assessments, detailed immunophenotyping including cytokine profiling, as well as relevant imaging and psychosocial parameters are warranted.

## Declaration of competing interest

This work was supported by 10.13039/501100004543China Scholarship Council (CSC) and the Department of Neurosurgery, 10.13039/501100005039Leiden University Medical Center (10.13039/501100005039LUMC), The Netherlands. Wensen Li received support from both funding agencies. The CSC and LUMC did not play a role in the design of the study, data collection or analysis of the data. For the remaining authors none were declared.
